# Examining the Effect of Contactless Intergenerational Befriending Intervention on Social Isolation Among Older Adults and Students’ Attitude Toward Companionship: Content Analysis

**DOI:** 10.2196/47908

**Published:** 2024-01-30

**Authors:** Keya Sen, Nida Laheji, Zo Ramamonjiarivelo, Cecil Renick, Randall Osborne, Brad Beauvais

**Affiliations:** 1 School of Health Administration Texas State University San Marcos, TX United States; 2 Department of Psychology Texas State University San Marcos, TX United States

**Keywords:** intergenerational befriending, social isolation, boredom, contactless socialization, service learning, internal motivation, mobile phone

## Abstract

**Background:**

Intergenerational friendship, a mechanism of social support, is an effective intervention to reduce the increasing risk of social isolation (SI) and develop companionship in the older adult population. The COVID-19 pandemic provided a unique opportunity to examine the psychosocial intervention of befriending via technology use as a primary form of contactless socialization.

**Objective:**

The study aims to explore the effectiveness of the befriending intervention through a contactless, intergenerational service-learning project on older adult emotions, especially boredom and loneliness as the key attributes of SI, and on students’ attitude toward companionship.

**Methods:**

During the months of January to April 2022 , undergraduate students enrolled in a health administration course with a special focus on culture were asked to be involved in a contactless, intergenerational service-learning project (n=46). In this study, contactless intervention meant communication using the telephone and apps such as FaceTime and Zoom. Students were paired with older adults to have at least a 30-minute weekly conversation, for 8 weeks, via telephone or an internet-based app such as FaceTime. Students were asked to write a half-page diary after each interaction and a 1-page reflection at the end of the fourth week and at the end of the service-learning project. At the completion of the project, the researchers also surveyed the older adults to assess the impact of the project using a 5-item open-ended questionnaire. Following a heuristic approach and content analysis, student artifacts (110,970 words; 118-page, single-spaced Microsoft Word document) and the older adult surveys were analyzed using MAXQDA, (VERBI GmbH). Qualitative data were extracted to assess the impact of service learning on SI by measuring the attributes of boredom and loneliness among 46 older adults. Students’ attitudes toward companionship were also assessed using data from their diaries and reflections.

**Results:**

Overall, three major constructs were identified: (1) meaningful engagement, defined as feeling safe, having increased confidence, and having reduced boredom; (2) internal motivation to participate in the weekly interaction, defined as discussion about daily life experience, level of happiness, and ability to exert personal control over the situation; and (3) intergenerational befriending, defined as perceived benefits from the friendly nature of the interaction, ability to comfortably connect with students, and positive feeling and attitude toward the student.

**Conclusions:**

The contactless, intergenerational befriending intervention reduced boredom and loneliness among older adults and enhanced positive attitude and confidence among university students. Students helped older adults to develop digital skills for the use of apps and social media. Older adults showed interest in the intervention and shared their daily life experiences with the students, which helped to reduce the gap between generations. Findings indicate the effectiveness of an intergenerational service-learning intervention on SI reduction and increased positive attitude among college students.

## Introduction

### Background

The growing surge of the aging population has shone a spotlight on social isolation (SI). With increasing age, an increasing proportion of older adults experience chronic boredom and feelings of loneliness [1]. In addition, COVID-19 confinement and quarantine disturbed their lifestyle behaviors, making the aging population susceptible to high levels of stress and depression. SI is exacerbated when people are living alone, especially vulnerable older adults, experiencing the loss of family or friends, having comorbid or chronic illnesses, and facing sensory impairments. Even in residential settings and older adults’ living facilities, many older adults struggle to maintain their autonomy, self-determination needs [2], and competence as they are often disconnected from the broad community or dependent on unskilled nursing staff who lack social competence and training [2,3]. An intergenerational service-learning approach to befriending these individuals is an effective intervention to enhance their psychosocial well-being [4]. This approach demonstrates the importance of using reflective writing assignments to help university students deconstruct and reconstruct images, beliefs, and paradigms about older adults [5]. In addition, it is where the young individuals relate to older adults to discuss or share their life stories or day-to-day experiences to alleviate boredom, an attribute of SI [6].

Although SI is a global epidemic implying the absence of meaningful human relations and social connectedness, befriending is a psychosocial tonic to building social relationships and intergenerational friendships [7] that might reduce boredom and depression and significantly enhance the quality of life among older adults [8] and youth. The feeling of companionship is augmented when an intergenerational conversation is conducted purposefully, thereby negating the exacerbating consequences of SI such as boredom or even the feeling of loneliness. These negative subjective experiences result from discrepancies between an individual’s desires and perceptions about the quality of social relationships [9].

Intergenerational conversations are a gateway to enduring socialization for older adults and students [9]. The quality of life is enhanced as both generations engage in purposeful activities in a safe and friendly environment [8] to the satisfaction of each other’s psychosocial needs and well-being. Long-lasting intergenerational conversations can create a bonding between the older adult and the student, and it enhances the level of patience and builds social competence in youth, which are essential attributes to become skilled health care professionals in long-term care [10]. The social support provided by students can foster emotional health for the isolated, frail, older adult who is no longer at the center of a network of friends and acquaintances. Older adults with social connections have a high quality of life under social support [11], are motivated to work and engage in social activities, and have an inclusive attitude that promotes a healthy lifestyle [12]. Hence, they have low risk of conditions such as depression, additive behaviors, or dementia [13]. With social connectedness, even in living alone situations, these individuals have life satisfaction, with high cognitive stability [10,14].

### Facets of SI

SI is one of the most disruptive transformations that exacerbate the quality of life, affecting human behavior, perceptions, and experiences [10,15]. It is a condition that leaves one with feelings of boredom and loneliness that are detrimental to physical and mental health [7]. Loneliness among older adults is the subjective, distressed feeling of being alone or separated, whereas SI is the objective physical separation from the community [14,16]. In addition, boredom is an emotion that often triggers negative thoughts such as self-harm and anxiety, and it is profound when the surrounding environment is mostly empty [17]. Hence, boredom, compounded by the feeling of loneliness over a prolonged period, may result in depression [18], which is a mental disorder that can be controlled in a timely way by identification of the needs of individuals and restoration of successful attention in meaningful activities such as intergenerational conversations. More than 56 million adults aged ≥65 years live in the United States, accounting for approximately 16.9% of the nation’s population [19]. In this population, >7 million (13%) are socially isolated. Of those 7 million individuals, 1.3 million are severely socially isolated [5,7]. The crucial drivers of boredom [18,20], namely, lack of recreational opportunities; limited personal contacts; immobility; prescribed home office; and, especially, COVID-19 quarantine and isolation have dissuaded the everyday lives of older adults, triggering anxiety and monotony [4,16,21].

Boredom across the population increased significantly owing to the COVID-19 pandemic containment measures [22], and so did the behavioral intention to find information, access services, and connect socially [23]. For older adults, there is evidential increase of the benefits of technology interventions for social connectedness [10,24]. This may have helped the contactless, intergenerational, service-learning intervention to become especially effective, facilitating the feeling of companionship and social support that is felt bilaterally and actively by both parties [25].

### Intergenerational Service Learning

Intergenerational service learning is an experiential learning, which is a course-based, credit-bearing, educational experience in which students participate in an organized, service-learning activity that meets identified community needs. Students reflect about the service activity to gain further understanding of a course content [26]. With an intent to develop a sense of companionship or friendship between both parties, the intergenerational service-learning intervention brings the socially isolated older adults back on the periphery of social activities as they engage in conversations with college students [27]. Recurrent interactive sessions are useful to enhance the level of subjective well-being among older adults and connect them to the social network [28].

Alternatively, the service-learning sessions provide a broad appreciation of discipline and an enhanced sense of civic responsibility to the students while connecting them in purposeful activity with community members [29]. Requesting students to write diaries and reflections regarding their interactions with older adult adults is a key component of service learning [25,29]. Although it is less likely to see older individuals readily embracing service-learning sessions involving students or even social media sites when compared with young adults, adoption rates for contactless interactions with students among individuals aged ≥65 years have approximately doubled in the United States in the past 4 years [14,30].

### Intergenerational Befriending Approach

This study used an approach that aims to bolster a long-lasting, genuine connection or relation between generations, especially college students engaged in service learning and older adults who are socially isolated [31]. This connection is based on shared experiences of daily living, which is reflected in the recurrent interaction based on the multicultural project. Although the intergenerational connection may develop over a contactless platform, it fosters a sentiment of compassion and empathy through focused interactions that add purpose and engagement for participants. The befriending idea is based on reciprocity and what benefits the older adult in a relationship [32]. The interactions are mostly based around topics that are meaningful to the older adult [10,33] and benefit the student to understand the biases of ageism. The idea is to make the older adult feel valued and cared for in a relation that is free from any kind of service delivery, obligation, or family ties [26,34].

### Contactless Socialization

The quality of life comprises components such as health; well-being; peaceful existence; living in harmony; social engagement; life satisfaction; and keeping oneself busy with hobbies, volunteer service, or work [8,35,36]. The older adults were subjected to a harsher reality during the pandemic than younger adults as old age was affirmed as a risk for COVID-19 complications [37]. This contention promoted contactless socialization through contactless service-learning sessions via SMS text message, FaceTime, and emails that connected the older adults to wide social networks [38]. Although user confidence remained as a dominant issue when using technology such as a smartphone or tablet for interaction needs [39], the willingness and interest of older adults let students train them on the use of technology. The training also facilitated the use of mobile health and telemedicine among older adults, which greatly enhanced their health-related quality of life [40]. Hence, technology-based, contactless, service-learning sessions provided safe interaction for both parties, fostering social support and technology skill augmentation for the older adults [41]. Simple telephonic calls were used to build intergenerational friendships [13,38] connecting student helpers with their clients through personal life experience, interpersonal interaction, collaboration, and understanding [42].

### Study Purpose

The study aimed to explore the effectiveness of the befriending intervention through a contactless, intergenerational, service-learning project on older adult emotions, especially boredom and loneliness as the key attributes of SI, and on students’ attitude toward companionship.

Our approach was to forge an alliance and create an affective bond between the young student and the older adult to facilitate the possibility of friendship as they shared their life experiences and students engaged in technology training activities for the older adults regarding the use of apps and social media sites. The relationship that develops through befriending is seen as central to the experience while hypothesizing the facts that befriending would foster psychosocial well-being among older adults and that students’ attitudes toward older adults would become more positive throughout the service-learning course.

## Methods

### Study Setting and Recruitment

The research design was based on qualitative data analysis [43]. Data were collected through the survey of older adults and dialogues included in journal entries submitted by 46 undergraduate students enrolled in a Health Administration course. This core or required course had a special emphasis on cultural competency and diversity. The study followed 46 older adults, aged between 64 and 82 years, via intergenerational service-learning sessions during the months of January to April 2022. The older adults were recruited from residential facilities in Good Samaritan Society, Denton, Texas; Schertz Senior Living, Texas; Knowles Home, Nashville, Tennessee; Aguadilla Seniors, Puerto Rico; and Guadalajara Senior Center, Mexico. We selected these locations because these are the largest centers for older adults known to the researchers, and we asked the older adults in these centers to invite other participants known to them to participate in the study, to expand the sample size.

The inclusion criteria for older adult participants consisted of the following: those who (1) were interested in socialization activities, (2) were aged >65 years, (3) could read English, and (4) were willing to participate in the study. The exclusion criteria were the following: older adults who (1) were aged <65 years, (2) were already engaged in >1 socialization activity, and (3) did not pass the “attention check” in a meeting with the researcher conducted before the intervention to assess their interests and identify careless respondents, thereby improving the data quality. The inclusion criteria of student participants were the following: all students enrolled in the health administration course irrespective of age or involvement in socialization activity. No exclusion criteria for students were determined.

### Data Collection Procedures

The service-learning sessions are an essential component of the undergraduate Health Administration course that has culture as a major topic. The course introduces undergraduate students to the historical and cultural development of health care in contemporary American society. During the months of January to April 2022 a total of 46 students were paired with 46 older adults from the abovementioned communities. Once a week, the students communicated with their assigned older adult partners and engaged in an unscripted conversation for at least half an hour, for a total of 8 weeks. Of the 46 pairs, 29 (63%) pairs engaged in telephonic conversations and 17 (37%) pairs engaged in internet-based conversations via casual calling app, such as FaceTime or Zoom. The risk of COVID-19 contamination restricted all possibilities of in-person meetings.

As part of service learning, students were required to create an artifact diary to document each conversation. Students were also asked to write a 1-page diary after each interaction with their older adult partners and 2 reflections. On the basis of the duration of the project, students were expected to write 8 diaries and 2 reflections, once at the end of their fourth interaction and then again at the end of their eighth interaction. The conversations were recorded by the students with their smartphones or technology used for internet-based interaction such as Zoom and then transcribed by the students.

As part of the study, older adults were surveyed (paper-based, 5-item open-ended questionnaire) by the researchers to determine their interest in conversation to reduce SI and evaluate the effectiveness of the project. This 1-time survey was mailed with return envelopes to the older adults at the end of the eighth interaction with students. Survey questions were open ended, so that older adults could write their answers. The students’ transcribed conversations, diaries, and reflections and the older adults’ answers to the open-ended questions in the survey were all used in the qualitative data analysis.

The open-ended survey questions for the older adults included the following: (1) Did you find the conversation interesting? If your answer is yes, please write a few lines what was interesting in the conversation. (2) Would you like to participate in our project again next semester?

The whole idea of the project was to provide a useful framework for befriending that may facilitate and create a meaningful bond between the young student and the older adult [44,45]. All questions were composed from previously validated survey instruments and contextualized for use in this study (refer to sources in Table 1). To assess older adult emotions, especially boredom and loneliness as the key attributes of SI, students focused on conversations (refer to definition in Table 1) based on life satisfaction and digital skill training in the context of meaningful engagement [46,47], internal motivation to participate in the intervention for both older adults and students [47-49], and human feelings in the context of intergenerational befriending or companionship [46,49,50]. Thus, in Table 1, we have presented 3 major constructs: meaningful engagement, internal motivation to participate, and intergenerational befriending.

**Table 1:** Study constructs, definitions, and sources.

Construct	Definition	Sources
Meaningful engagement	Frequency of socially interactive activities Feeling of reduced boredom Cohesive interaction (confidence and safety)	Questions about satisfaction with life, contextualized from the studies by Diener et al [46] and Gierveld and Tilburg [47]
Internal motivation to participate	Discussion about daily life experience Level of happiness Ability to exert personal control over the situation	Questions about social isolation and motivation, contextualized from the studies by Kozma and Stones [48], Gierveld and Tilburg [47], and Russell [49]
Intergenerational befriending	Perceived benefits from the friendly nature of interaction that develops mutual trust Ability of the older adults to comfortably connect with students Positive feeling and attitude of the students	Questions about human feelings, contextualized from the studies by Diener et al [46], Russell [49], and Golden et al [50]

### Ethical Considerations

This contactless, intergenerational, befriending interventional study was approved by the institutional review board (protocol number 2022-7046) of the Texas State University. Written informed consent was obtained from all participants (students and older adults) before the intervention. Participants had the option to exit the intervention after reading the informed consent information or to provide consent to participate in the study. The confidentiality of the participants was properly protected during the intervention and data analysis. The study data were fully deidentified. All records pertaining to the intervention were securely protected in the university database with protected passwords, which were only accessible by the researchers. As this study was not grant funded, participants (undergraduate students and older adults) were not compensated for participating in this study.

### Data Analysis

The older adults’ survey was mailed to the 46 older adult participants, and 21 (46%) returned the completed survey. Students’ diaries and reflections were collected at the end of the intervention (46/46, 100%), and all the files were used for data analysis. The qualitative data used in the study to extract the constructs and subconstructs were obtained from the 21 older adults’ surveys and 46 students’ diaries and reflections. Our analytic data included all the 110,970 words in a 118-page, single-spaced Microsoft Word document.

Following a heuristic approach [51] and content analysis, the data were coded using the qualitative text analysis software, MAXQDA by numbering each line of the dialogue [52]. The “Advanced Coding Method” in MAXQDA Standard was used for content analysis with major keywords that were allocated to data segments. The “Lexical Search” function located the keywords in all the text that define the 3 major constructs as identified in Table 1. Once the keywords of befriending, SI, boredom, socialization, engagement, and motivation were identified, the thematic coding of the relevant texts was performed using MAXQDA’s visual tool, “One Code Model.” We explored the frequency of words and terms used in the sources and analyzed their semantic contexts in a quantitative way. A differentiated word frequency analysis was performed using the “MAXDictio Module” [52]. The data were then classified into several groups to reveal trends and patterns of response to each question in the survey and in each topic of conversation between the older adults and students from the student dairies and reflections. Topically similar codes were grouped together and then narrowed by code segments using the option “Subcode Statistics” in the context menu of the “Code System” of MAXQDA. For example, the code “intergenerational befriending” was further subcoded as “comfortability,” “positive emotions,” and “perceived benefits.” Finally, using the MAXQDA function “Analysis Summary Grid,” thematic compilations were presented in “Participant Comment Tables.”

The use of heuristic inquiry [51,52] helped us to discover the nature of social phenomena, especially the intergenerational bonding that developed between participants, as we systematically coded the data. Owing to the complex nature of the emerging themes related to befriending and self-expression, heuristic inquiry appeared to be the most convenient method of sense making for this study. The heuristic depictions of the artifacts involved a synthesis of intuition and tacit understanding of researchers [53]. This understanding characterized the idea of befriending experience. The concepts that emerged from the study included relationship building, empathy, social interactions, and capacity for additional relational networks outside the family.

## Results

In the older population of 46 adults, of which 19 (41%) were men and 27 (59%) were women, 3 main constructs were identified: intergenerational befriending, meaningful engagement, and internal motivation to participate in the program. According to the older adult participants, the perceived benefits of the friendly nature of intergenerational interaction were mainly the heightened feelings of comfort and reduced boredom. For both the student and older adult participants, trust in the interactions bolstered the positive emotions, enhancing the feeling of safety and social affinity. Table 2 displays the number of times the older adults’ and student participants’ specifics about the scope of befriending and engagement to reduce boredom. Meaningful engagement was described as “reduced boredom,” “feeling safe,” and “increased confidence.” For a total of 253 times, the older adult participants mentioned that they were purposefully engaged, 96 times they reiterated their feeling of happiness, 58 times about feeling safe, and 98 times that there was an impressive increase in their level of confidence. Similarly, students mentioned 201 times that they were meaningfully engaged in the project, 91 times they felt happy, 55 times that they did not feel bored in the conversations with older adults, and 97 times about the increased level of confidence.

Intergenerational befriending was summarized as “comfortability” and “positive emotions.” The older adult participants specified 97 times that they benefited from the program and that they made new friends. For many older people, “engagement to reduce boredom” specified 97 times, was the main reason for participating in these conversations. Intergenerational sessions enhanced the motivation level of the older adults 277 times, with improvement in emotional health or positive emotions (specified 80 times). Having company was more of an antidote to reduce boredom (specified 90 times). The discussions about daily life experience led to emotive bonding and friendship. For the students, the befriending experience (specified 201 times) enhanced the feeling of comfort (specified 70 times) and positive emotions (59 times). Both the students and older adult participants looked forward to the sessions and considered it as a reason to get up in the morning.

The intergenerational service-learning intervention mainly covered two activities: (1) discussions about daily life experience to reduce the gap between generations and (2) digital training to help older adults to use social media sites and apps such as Facebook or Uber. The main attributes of SI targeted in this study, namely, boredom and loneliness, were minimized to some extent with the befriending approach as people felt safe and happy as they interacted with the students. Importantly, older adults were extremely interested in the technology training from students regarding how to use social media sites and apps such as Facebook and Uber. Tables 3 and 4 reflect the confirmatory statements of the older adults and students toward the valuable “befriending” component that engaged older adult participants substantially with the undergraduate college students in digital training and motivated them to share their life experiences safely. The attributes of hesitation and fear, which were noticed in the initial stages, turned to compassion, trust, respect, empathy, honesty, and warmth in the later stages, which are the most essential attributes of friendship.

The positive attitude and the dedication of the students toward the project helped the older adults to find social support and feel safe. On most occasions, activities were based upon the interest of the older adults. The opportunities for social interaction were possible as students were proactive and flexible with time and the needs of their older adult partners. The results revealed the importance of students’ positivity to support the older adults and the increased level of motivation for spontaneous interactions. The students created a friendly environment, or that of companionship, where the older adults had confidence that the discussions were appropriate based on their choices and preferences.

Another key finding was that older people define boredom and comfort differently and that there is a difference between the students’ perceptions and older adults’ perceptions related to interactions to alleviate boredom. Although befriending through these sessions cannot compensate for the loss of an attachment figure, such as a close friend, spouse, or a significant other, which is common in old age, people with poor social skills are likely to have trouble in developing and maintaining relationships. Negative perceptions about age and aging, at societal and individual levels, have adverse effects on older adults’ health and well-being. Ageism, which means negative attitudes toward older adults, or unrealistic expectations about the intergenerational sessions can leave both the older adults and the young individuals with unmet social needs, resulting in increased boredom. Hence, the befriending approach must be understood from the individual’s subjective point of view.

**Table 2:** Constructs and subconstructs identified from the intergenerational service-learning intervention.

Name of the constructs and subconstructs			Number of times specified by older adults (n=786), n (%)		Number of times specified by students (n=557), n (%)
**Intergenerational befriending**			256 (32.6)		179 (32.1)
	Comfortability	79 (10.1)		70 (12.6)	
	Positive emotions	80 (10.2)		59 (10.6)	
	Perceived benefits	97 (12.3)		50 (8.9)	
**Internal motivation**			277 (35.2)		177 (31.8)
	Feeling of happiness	96 (12.2)		91 (16.3)	
	Discussion about daily life experiences	181 (23)		86 (15.4)	
**Meaningful engagement**			253 (32.2)		201 (36.1)
	Feeling safe	58 (7.4)		49 (8.8)	
	Increased confidence	98 (12.5)		97 (17.4)	
	Reduced boredom	97 (12.3)		55 (9.9)	

**Table 3:** Comments of older adults about the key constructs.

Key construct	Comments
Meaningful engagement	“Like to talk about you and the multicultural sensitivity project in the Zumba classes three times a week, and ballet classes twice a week.” [Participant 2] “Feel glad, I seem to have more control on anger unlike before, we have opportunities for social interaction and there is less counterproductive behavior.” [Participant 45] “She plays for the university’s basketball team. Since the university is so close to where I live, I get to attend all her games and is eternally grateful for this.” [Participant 5] “The student group hosts a couple of social events every day, so there is always an event to attend and enjoy. Some of these events include- bingo, bible studies, stretching class, and physical fitness classes as well.” [Participant 42] “She is physically very active, I enjoyed our zoom discussion last week, me and my husband, engaging in either yoga, meditation, or going on 30-minute walks.” [Participant 16]
Internal motivation to participate	“Family reunion was over; it was a good day of our weekly conversation and i-phone training.” [Participant 39] “Grateful to be able to wake up each morning and do the things that I enjoy, like art and seeing family and talking to my student friend over the phone.” [Participant 3] “Like to talk to you about school, do you enjoy the simple pleasures in life like cooking?” [Participant 28]
Intergenerational approach to befriending	“Now look forward to our weekly conversations and app training sessions.” [Participant 40] “I don’t get to see my grandchildren as often as I would like, so having a person of the younger generation to talk to weekly reminds me a lot of them.” [Participant 25]

**Table 4:** Comments of students about the key constructs.

Key construct	Comments
Meaningful engagement	“At first the project was challenging, towards the end I felt relaxed, as I came to know the older adult partner more closely.” [Participant 9] “She explained it so well, I never thought before that smoothie making could be so simple.” [Participant 11]
Internal motivation to participate	“She loves everything about her life. Our hobbies are the same. She is blessed to be healthy at her age, and she wouldn’t change a thing.” [Participant 12] “My grandmother used to tell me similar things, to be able to wake up each morning and enjoy breakfast with family.” [Participant 1]
Intergenerational approach to befriending	“Feels nice to be in an interesting conversation and write diaries.” [Participant 46] “So happy to help someone who sees her grandchild in me.” [Participant 45]

## Discussion

### Summary

The befriending intervention delivered through the service-learning sessions promoted reciprocity and social support that enhanced the quality of life by fostering positive emotions and reduced boredom and loneliness. The intergenerational service-learning program fostered discussions about daily life experience that reduced the gap between generations and promoted companionship [54]. The proactiveness and dedication of the students for their project helped the older adults to find social support and feel safe and created a bonding between the generations with long-lasting friendships.

### Meaningful Engagement

Through this specific intergenerational service-learning experiment, we found that most older adults try to keep themselves occupied through personal hobbies; talking to family over the phone; and occasionally, even work. Meaningful engagement was depicted well through the comments of older adult participant 42 who was happy and well engaged in programs and activities administered by the university such as telephone-based befriending and contactless luncheons with students. For these participants, social interaction through the service-learning program enhanced connectivity and reduced the risk of losing the motivation to maintain an active and healthy lifestyle. The conversations reduced the feelings of boredom and isolation that adversely affected their emotional health. These sessions helped the older adult to reengage in community networks and participate freely in cohesive neighborhood environments with other members of the older adult living facility. Older adult participant 2 found these sessions to be meaningful and interesting, explained the importance of these sessions to friends and community, and constantly spoke about the interactions in the Zumba and ballet classes 2 to 3 times a week. The intergenerational service-learning conversations toward the end of the project showed a heightened level of comfort for both the older adults and the youths and made the older adults feel that they are cared for while in conversation. Hence, they were not bored in their conversation with the students.

### Internal Motivation to Participate

For the older adults, there is the need of a companion to share life experiences [55], which is profound when separated from the family owing to relocation, death of family members, or retirement [56]. The befriending activity reduced the boundaries between the 2 people involved, and the student befriender was asked to undertake tasks that the older adult wanted to do, such as using technology or having contactless visits during family reunion. This created a sense of internal motivation for older adult 40 to participate in the interactions. As students helped the older adults to interact with their family and share life stories, these interactive sessions fostered companionship that was of interest to the older adult and a reason for the student to find meaning and value in the conversations. ​Following the comments of older adult participant 25 and student participant 45, we found discussions about spouses to children and grandchildren and, for some, even to great grandchildren. All familial connections showed the importance of social support and motivated both parties in conversation on a day-to-day basis. Student participant 12 prayed for the well-being of his older adult partner and felt happy as he discovered a common hobby to talk about. Older adult participant 39 described her spouse returning from rehabilitation and mentioned that a welcome home party with her spouse’s family resulted in her having a good day. Older adult participant 5 revealed that she is eternally grateful to be able to attend her befriender’s basketball games; the joy and motivation to be able to socially connect seemed to have a positive effect on her well-being.

### Intergenerational Approach to Befriending

Although several of the older adults in this study had various outlets to socialize such as hobbies, calling a friend, and family visits outside the intergenerational service-learning sessions, they still expressed appreciation toward the students for their time and companionship, which fulfilled the idea of befriending that we aimed for in this study. Student 45 noted that the older adult was nervous at the beginning but eventually looked forward to their weekly conversations, further mentioning that having someone from the young generation to talk to reminded her about her grandchildren whom she does not get to see often. In another instance, student participant 46 would write down events worth mentioning every week to discuss during the call. The overall evaluation of the intergenerational conversation was positive; older adult 40 wrote in the survey that she valued the ability to talk, listen, and share information with another human being whom she could trust and rely on. The befriending approach within these sessions appeared to expose the similarities between 2 different generations as the weeks passed. For example, student 28 revealed that he felt lucky to share the same religious and spiritual beliefs as the older adult partner. Similarly, student 42 stated that she and her older adult partner shared the same type of church. Most of the student’s reflections revealed the idea of reliability, compatibility, intimacy, and reciprocity in the conversations, which had a positive effect on promoting friendship and reducing boredom and loneliness for both parties. In addition, the program improved the youth’s social skills and assumptions related to one’s privacy and safety.

### Limitations

This study has some limitations. Several students reported being unable to communicate with their partners at the beginning of the project owing to difficulty with older adults’ digital literacy, inability to answer phone calls or read SMS text messages, or inability to use apps such as Zoom or FaceTime. This ruled out the possibility of Zoom focus groups. In-person focus groups were also not possible owing to COVID-19 socialization restrictions. In some cases, there was dropout of older adults from the project owing to sickness or other health conditions. Although the authors clearly explained to the older adults that the service-learning project was completely contactless, some of them still preferred face-to-face interaction, which was not possible because of COVID-19 restrictions. Furthermore, student notes may not be free from bias. The findings from this study have limited generalizability because we used a sample of students from 1 course, and the results may not be applicable in other study settings. Variables such as overall health, stress, stigma, comorbid conditions, socioeconomic status, and discrimination were not considered while assessing older adult surveys or student artifacts in the study.

### Conclusions

The need to feel important and be included in a large social group is vital regardless of age. The need is perhaps more during the later stages of life when one has more physical and mental challenges and is confined in living situations with very limited social networks. The effects of service-learning sessions on boredom and loneliness among older adults promoted interest in intergenerational practice to reduce SI and improved the feelings of companionship among both students and older adults as a long-term outcome of the intervention. Students identified the intergenerational component as a highlight of the health administration course, which increased their awareness of gerontological issues and knowledge about working with aging populations. The intervention enhanced students’ attitude toward companionship and gerontological practices via intergenerational befriending opportunities and digital training sessions. As students developed an interest in service-learning programs through frequent interactions with older adults, the social interaction component in the program amplified social connectivity via contactless interactions, phone conversations, and digital training sessions for older adults. Consequently, it reduced the risk of losing the motivation to maintain a self-image and an active and healthy lifestyle among older adults. This study was designed for periodic or postpandemic follow-up of the participants experiencing SI. Our results showed that older adults wished to maintain their social connectedness and access to technology and digital networks. Driven by a desire to communicate and access information that stemmed from the need to connect to friends, family, or other internet-based services such as mobile health or social media, older adults showed appreciation and gratitude toward the youth for taking an interest in their lives and, often, would look forward to their weekly conversations.

## Abbreviations

SI: social isolation

## Data Availability

The institutional review board of this study does not allow data sharing because the data protection statement is an integral part of our institutional review board approval. Therefore, our data will not be made available.
